# Identification of Novel Pathogenic Variants in Familial Adenomatous Polyposis Through Whole Genome Sequencing

**DOI:** 10.1155/humu/1896928

**Published:** 2026-08-19

**Authors:** Sung-Ryung Choi, Min Jung Kim, Ja-Lok Ku, Seung-Yong Jeong, Murim Choi, Ji Won Park

**Affiliations:** ^1^ Department of Translational Medicine, Seoul National University College of Medicine, Seoul, Republic of Korea, snu.ac.kr; ^2^ Department of Surgery, Seoul National University College of Medicine, Seoul National University Hospital, Seoul, Republic of Korea, snuh.org; ^3^ Cancer Research Institute, Seoul National University College of Medicine, Seoul, Republic of Korea, snu.ac.kr; ^4^ Department of Biomedical Sciences, Seoul National University College of Medicine, Seoul, Republic of Korea, snu.ac.kr; ^5^ Ischemic/Hypoxic Disease Institute, Seoul National University Medical Research Center, Seoul, Republic of Korea, snu.ac.kr

**Keywords:** aberrant splicing, *APC*, exon skipping, familial adenomatous polyposis, pseudoexon activation, whole genome sequencing

## Abstract

Familial adenomatous polyposis (FAP) is an autosomal dominant colorectal cancer predisposition syndrome most commonly caused by pathogenic variants in the *APC* gene. Although targeted gene panel sequencing is routinely used, some FAP cases remain genetically unresolved due to limitations in detecting structural and deep intronic variants. We investigated two unrelated kindreds with clinically evident but genetically unexplained FAP using targeted panel sequencing and whole exome sequencing. Pedigree‐based whole genome sequencing (WGS) was performed to detect cryptic variants, with in silico splice prediction and transcript‐level validation using RT‐PCR, Sanger sequencing, and RT‐qPCR of blood‐derived RNA. WGS uncovered two novel pathogenic *APC* variants missed by prior testing. In Kindred 1, a heterozygous large deletion encompassing exon 10 was detected and confirmed to cause exon skipping, resulting in a frameshift and premature termination codon. In Kindred 2, a deep intronic complex deletion‐insertion variant was identified between exons 11 and 12, predicted to create novel splice sites. Transcript analysis demonstrated aberrant pseudoexon activation with a 2‐base‐pair deletion, leading to a frameshift and premature termination. Both variants segregated with disease within the respective families and were classified as pathogenic according to ACMG guidelines based on loss‐of‐function effects and functional RNA evidence. Our findings demonstrate that cryptic pathogenic *APC* variants beyond the detection limits of routine genetic testing can be resolved through WGS combined with transcript‐level validation. This study highlights the importance of transcript‐aware variant interpretation and supports the integration of genome‐wide sequencing and RNA‐based analyses into the diagnostic evaluation of genetically unexplained FAP.

## 1. Introduction

Familial adenomatous polyposis (FAP) is a rare but well‐defined autosomal dominant disorder, accounting for less than 0.5% of all colorectal cancer cases [1]. Clinically, it is marked by the emergence of hundreds to thousands of adenomatous polyps throughout the colon and rectum, typically beginning in the second decade of life. Without prophylactic surgical intervention—most commonly total proctocolectomy with ileal pouch–anal anastomosis (TPC‐IPAA) or colectomy with ileorectal anastomosis—affected individuals carry a near‐100% lifetime risk of colorectal cancer, often by the fourth decade of life [2–4]. Diagnosis is typically established by colonoscopic identification of characteristic polyposis, with genetic confirmation via adenomatous polyposis coli (*APC*) variant analysis. In addition to colorectal disease, FAP is associated with a range of extracolonic manifestations, including desmoid tumors, congenital hypertrophy of the retinal pigment epithelium (CHRPE), osteomas, and upper gastrointestinal polyps, which contribute to overall disease morbidity and inform long‐term surveillance strategies.

Approximately 90% of FAP cases are caused by highly penetrant heterozygous pathogenic variants in the *APC* gene located on chromosome 5q22 [5]. The *APC* gene (OMIM 611731) spans approximately 100 kb of genomic DNA and encodes a large multifunctional tumor suppressor protein of 2,843 amino acids. The canonical *APC* transcript (MANE Select: NM_000038.6; ENST00000257430.9) comprises 16 exons, with the final coding exon accounting for the majority of the protein‐coding sequence and representing a mutational hotspot [6]. APC is ubiquitously expressed, with particularly high levels in proliferative epithelial compartments such as the intestinal crypts, where it plays a critical role in maintaining tissue homeostasis.

At the protein level, APC functions as a scaffold protein harboring multiple functional domains, including an N‐terminal oligomerization domain, armadillo repeats mediating protein–protein interactions, 15‐ and 20‐amino acid repeats responsible for *β*‐catenin binding, SAMP repeats that interact with AXIN, and C‐terminal domains involved in cytoskeletal regulation. Through these domains, APC forms a *β*‐catenin destruction complex with AXIN, glycogen synthase kinase 3*β* (GSK3*β*), and casein kinase 1 (CK1), promoting phosphorylation and proteasomal degradation of *β*‐catenin. Loss‐of‐function variants in *APC* disrupt this regulatory complex, resulting in nuclear accumulation of *β*‐catenin and constitutive activation of Wnt target genes, ultimately driving uncontrolled cellular proliferation and colorectal tumorigenesis [7, 8]. Beyond its canonical role in Wnt/*β*‐catenin signaling, APC interacts with multiple proteins—including EB1, DLG, and components of the microtubule network—and is involved in cytoskeletal organization, cell migration, chromosomal segregation, and mitotic spindle assembly, thereby contributing to the maintenance of genomic stability [9, 10].

Aberrant activation of the APC–Wnt/*β*‐catenin pathway also underlies extracolonic manifestations such as desmoid tumors, providing a biological rationale for the use of nonsteroidal anti‐inflammatory drugs in their clinical management [11–14]. Well‐established genotype–phenotype correlations further inform clinical decision‐making: truncating variants within the mutation cluster region (MCR; codons 1250–1450) are typically associated with classic FAP characterized by profuse polyposis, whereas variants near the 5 ^′^ or 3 ^′^ ends of the gene or certain splice‐altering variants are associated with attenuated familial adenomatous polyposis (AFAP), which presents with fewer polyps and later disease onset. Specific variant locations have also been linked to desmoid tumors and CHRPE, reflecting domain‐specific functional consequences of APC disruption. To date, thousands of *APC* variants have been reported in public databases, including numerous pathogenic truncating and splice‐altering alleles associated with FAP [4, 15–17].

Despite the widespread implementation of targeted gene panel sequencing and whole exome sequencing (WES) in clinical diagnostics, around 10%–30% of patients with clinically suspected FAP remain genetically unresolved [18, 19]. This diagnostic gap is partly attributable to the technical limitations of exome‐based approaches in detecting certain classes of variants, including exon‐level deletions, complex structural variants, and deep intronic splice‐altering variants [20–22]. In particular, regions of transcript complexity, variable exon usage, or insufficient capture coverage may hinder reliable variant detection and interpretation. Recent studies have demonstrated that transcriptome‐based analyses can substantially improve diagnostic yield in WES‐negative cases by directly identifying aberrant splicing events and cryptic exon activation that are missed by exome sequencing alone [23–25].

Large exon‐level deletions within *APC* are considered relatively uncommon and are likely under‐recognized, especially when breakpoints reside within intronic regions that are poorly captured by WES. Moreover, the increasing recognition of transcript diversity—including differences between the MANE Select transcript and minor isoforms—has added complexity to variant interpretation, particularly for splice‐altering variants that may not affect the canonical transcript directly. Deep intronic variants capable of activating cryptic splice sites or pseudoexons represent an additional diagnostic challenge, as their pathogenic effects are often missed without transcript‐level analysis [26, 27].

In this study, we report two independent families with clinically diagnosed FAP in whom routine genetic testing failed to identify pathogenic variants. Using pedigree‐based WGS, we identified two distinct cryptic pathogenic *APC* variants: (i) a novel heterozygous large deletion of ~4 kb encompassing exon 10 and (ii) a deep intronic complex deletion‐insertion (delins) variant that induces aberrant splicing through pseudoexon activation. Both variants were functionally validated at the RNA level using reverse transcription PCR (RT‐PCR), Sanger sequencing, and RT‐qPCR, demonstrating frameshift events leading to premature termination codons. Our findings highlight the critical role of WGS combined with transcript‐level functional validation in resolving genetically unexplained FAP cases and underscore the importance of transcript‐aware variant interpretation in the molecular diagnosis of APC‐associated disease.

## 2. Materials and Methods

### 2.1. Family Screening and Clinical Data Collection

Detailed family histories were obtained through structured interviews with the proband and family members. Clinical data, including colonoscopy results and histopathological findings, were retrieved from the Hospital Information System (HIS) of Seoul National University Hospital (Table 1). Peripheral blood samples were collected from available family members for germline *APC* variant screening and transcript analysis. This study was approved by the institutional review board (IRB) of Seoul National University Hospital (No. 1103‐125‐357), and all participants provided written informed consent prior to enrollment.

**Table 1:** Summary of affected individuals from Kindred 1 and Kindred 2.

	Kindred 1		Kindred 2		
Feature	IV‐5 (Proband)	IV‐10	III‐2 (Proband)	III‐3	III‐4
Sex	Female	Male	Male	Female	Male
Age at diagnosis (years)	20	20	31	28	29
Colorectal polyposis	Hundreds of adenomatous polyps (0.5–2.0 cm), pan‐colonic		Numerous adenomatous polyps, pan‐colonic		
Histopathology	> 100 tubular adenomas, no malignancy	Multiple tubular adenomas, low‐grade dysplasia	> 100 tubular adenomas, no malignancy		
Surgical treatment	Laparoscopic TPC‐IPAA (1–12 years after diagnosis)				
Extracolonic manifestations	Desmoid tumors (> 10 cm, postop); SPINK1‐related recurrent pancreatitis; gastric polyposis	Gastric polyposis	Gastric polyposis	Not available	Not available
Family history	II‐1, II‐3: CRC (deceased at 60, 54 years); III‐5, III‐6, III‐8: FAP + CRC (surgery at 27, 30, 31 years)	Same kindred as IV‐5	Maternal grandmother: CRC (deceased ~60 years); mother and maternal aunt: CRC (early 40s, surgery); maternal aunt′s children: FAP (late 20s, surgery)	Same kindred as III‐2	

Abbreviations: CRC, colorectal cancer; TPC‐IPAA, total proctocolectomy with ileal pouch–anal anastomosis.

### 2.2. Genomic Analysis

**Figure 1:**
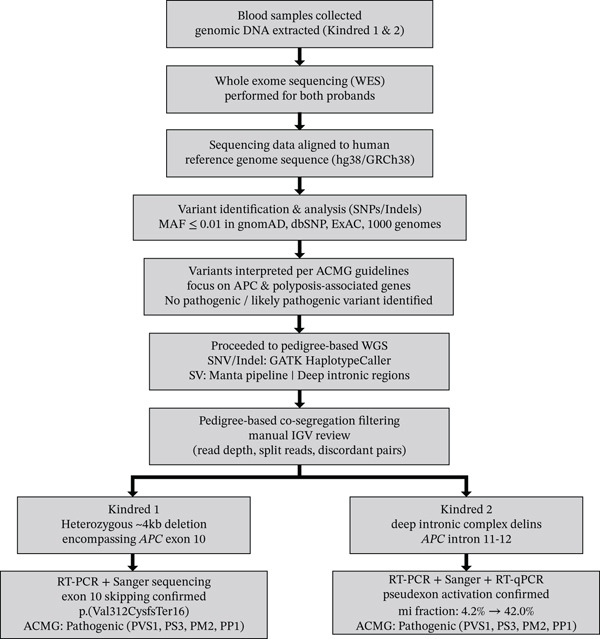
Variant identification and functional validation workflow. Schematic overview of the diagnostic and analytical pipeline applied to Kindred 1 and Kindred 2. Following nondiagnostic whole exome sequencing (WES), both kindreds underwent pedigree‐based whole genome sequencing (WGS). Small variants were called using GATK HaplotypeCaller, and structural variants were detected using the Manta pipeline. Candidate variants were prioritized by cosegregation filtering and manual IGV review. In Kindred 1, a heterozygous ~4 kb deletion encompassing *APC* exon 10 was identified and confirmed by RT‐PCR and Sanger sequencing (p.(Val312CysfsTer16); pathogenic: PVS1, PS3, PM2, PP1). In Kindred 2, a deep intronic complex delins variant in *APC* intron 11–12 was identified, with pseudoexon activation confirmed by RT‐PCR, Sanger sequencing, and RT‐qPCR (mi fraction: 4.2% → 42.0%; pathogenic: PVS1, PS3, PM2, PP1). MAF, minor allele frequency; SV, structural variant; IGV, Integrative Genomics Viewer.

Genomic analysis was performed in individuals IV‐5 and IV‐10 from Kindred 1 and III‐2, III‐3, and III‐4 from Kindred 2. Genomic DNA was extracted from peripheral blood leukocytes using the Exgene Blood SV Mini Kit (GeneAll, Seoul, Korea) according to the manufacturer′s protocol and stored at −20°C until further analysis. Affected individuals initially underwent WES as part of routine clinical genetic testing. Because no pathogenic or likely pathogenic variants were identified, WGS was subsequently performed for further investigation (Figure 1).

Variant prioritization focused on genes associated with colorectal polyposis, including *APC*. Raw sequencing reads were aligned to the human reference genome (GRCh38/hg38) using BWA‐MEM. Duplicate reads were marked using Picard MarkDuplicates, and base quality score recalibration (BQSR) was applied to improve variant calling accuracy (Table S1). Small variants were called using GATK HaplotypeCaller, and structural variant analysis was performed using the Manta pipeline. Variants were filtered based on quality metrics including depth of coverage, genotype quality, and allele balance. Pedigree‐based filtering was applied, and only variants segregating with the disease phenotype within each family were considered. Rare variants were prioritized using population frequency data from gnomAD (minor allele frequency < 0.01) and in silico pathogenicity prediction tools. Candidate variants were manually reviewed using the Integrative Genomics Viewer (IGV) to evaluate read‐depth changes, discordant paired‐end reads, and split‐read evidence suggestive of structural variation. This analytical framework follows previously described approaches for WES‐ and WGS‐based rare variant identification in inherited disease [28–30].

### 2.3. Transcript Analysis

Transcript analysis was performed in Individuals III‐5, III‐8, IV‐5, IV‐10, and IV‐11 from Kindred 1 and III‐2, III‐3, and III‐4 from Kindred 2. Total RNA was isolated from peripheral blood samples using the Hybrid‐R Blood RNA Kit (GeneAll, Seoul, Korea) following the manufacturer′s instructions. Complementary DNA (cDNA) was synthesized from total RNA using oligo(dT) primers and the AccuPower RocketScript Cycle RT Premix (Bioneer, Daejeon, Korea).

For transcript analysis in Kindred 1, a 601‐base‐pair (bp) region spanning exons 9–11 of *APC* was amplified to assess exon‐level alterations using the following primers:

• Forward: 5 ^′^‐TCTGGTAATGGTCAGGGTTCA‐3 ^′^.
• Reverse: 5 ^′^‐CCTGTAGTCCCCCTAGTTCAT‐3 ^′^.

PCR amplification was performed under a standard condition, and products were separated by electrophoresis on a 1.5% agarose gel. Aberrant bands were excised, purified, and subjected to bidirectional Sanger sequencing to characterize exon‐level alterations.

For transcript analysis in Kindred 2, primers were designed to amplify the region spanning *APC* exons 11–12 to evaluate aberrant splicing. The primer sequences were as follows:

• Forward: 5 ^′^‐GCCAGCTCCTGTTGAACATC‐3 ^′^.
• Reverse: 5 ^′^‐GCCATTCCAGCATATCGTCT‐3 ^′^.

Based on transcript structure, the expected amplicon size for the canonical *APC* transcript was 198 bp, whereas the corresponding region in a known minor *APC* transcript isoform (ENST00000504915.2) measured 252 bp. In affected individuals, activation of cryptic splice sites resulted in the inclusion of an aberrant exon with a 2‐bp deletion, producing a 250‐bp amplicon. PCR products were separated on a 2.5% agarose gel. Distinct bands corresponding to canonical, minor, and aberrant transcripts were visualized, excised, and subjected to bidirectional Sanger sequencing.

### 2.4. Sanger Sequencing

Sanger sequencing was performed to validate the transcript‐level findings identified by RT‐PCR. The same primer pairs used for RT‐PCR amplification were employed for sequencing in both kindreds (Kindred 1: forward 5 ^′^‐TCTGGTAATGGTCAGGGTTCA‐3 ^′^, reverse 5 ^′^‐CCTGTAGTCCCCCTAGTTCAT‐3 ^′^; Kindred 2: forward 5 ^′^‐GCCAGCTCCTGTTGAACATC‐3 ^′^, reverse 5 ^′^‐GCCATTCCAGCATATCGTCT‐3 ^′^). Sequencing chromatograms were analyzed and aligned to the reference *APC* transcript (NM_000038.6; MANE Select) to determine exon skipping events and precise splice junctions. Predicted protein‐level consequences, including frameshift events and premature termination codons, were assessed based on the same reference sequence.

### 2.5. RT‐qPCR

Quantitative reverse transcription PCR (RT‐qPCR) was performed to assess the relative abundance of the major and minor *APC* transcripts in Kindred 2. All reactions were carried out using TOPreal SYBR Green Premix (Enzynomics, Daejeon, Korea) on a QuantStudio 3 Real‐Time PCR System (Applied Biosystems, Foster City, California, United States).

Each 20 *μ*L reaction contained 10 *μ*L of 2× TOPreal SYBR Green Premix, 2 *μ*L of forward and reverse primer mix (5 *μ*M each; 300 nM final concentration per primer), and 8 *μ*L of cDNA template. All reactions were performed in duplicate. A master mix containing TOPreal SYBR Green Premix and the respective primer pair was prepared in advance for each of the three primer sets (*GAPDH*, major (MA) transcript‐specific, and minor (mi) transcript‐specific), with a 10% excess volume to account for pipetting loss. The master mix was dispensed into PCR tubes prior to cDNA template addition. Tubes were vortexed for at least 30 s and briefly centrifuged to eliminate air bubbles before loading.

Thermal cycling was performed under the following conditions: initial polymerase activation and denaturation at 95°C for 10 min; 40 cycles of denaturation at 95°C for 10 s, annealing at 62°C for 15 s, and extension at 72°C for 30 s; followed by melt curve analysis from 55°C to 95°C in 0.5°C increments. *GAPDH* was used as a reference gene for normalization. The transcript ratio between the MA and mi *APC* isoforms was quantified within each sample using the 2^(−*Δ*Ct)^ method, with *Δ*Ct calculated as the difference between the target Ct and the *GAPDH* Ct. Primer sequences are provided in Table S2.

## 3. Results

### 3.1. Clinical Characteristics of Two Families

**Figure 2:**
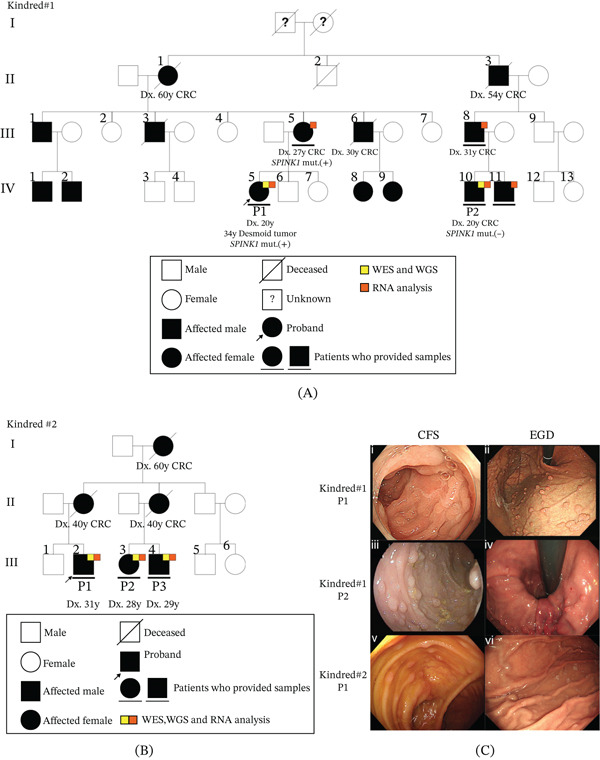
Clinical characteristics and pedigrees of two families with FAP. (A) Pedigree of Kindred 1 showing autosomal dominant inheritance of FAP. Filled symbols indicate affected individuals, and open symbols indicate unaffected individuals. Underlined symbols indicate individuals who provided biological samples. The proband (P1) and her second cousin (P2) underwent WGS following nondiagnostic WES. Transcript‐level analysis was performed in P1, P2, and three affected individuals (III‐5, III‐8, and IV‐11). Age at diagnosis and history of colorectal cancer are indicated below each symbol. A pathogenic *SPINK1* variant (c.194+2T>C) was identified in P1 and individual III‐5. (B) Pedigree of Kindred 2 demonstrating segregation of the disease phenotype across generations. The proband (P1) and two additional affected individuals (P2 and P3) underwent WGS following nondiagnostic WES. (C) Representative endoscopic findings from affected individuals. Colonoscopy (CFS, left) and esophagogastroduodenoscopy (EGD, right) images are shown for Kindred 1, individual P1 (i and ii), Kindred 1, individual P2 (iii and iv), and Kindred 2, individual P1 (v and vi), demonstrating numerous adenomatous polyps consistent with classical FAP.

Both families presented with clinical features consistent with FAP. The overall diagnostic and analytical workflow is summarized in Figure 1. Affected individuals demonstrated hundreds of colorectal adenomatous polyps on colonoscopic examinations, with disease onset in early adulthood. Pedigree analysis revealed autosomal dominant inheritance patterns in both families (Table 1, Figure 2A,B).

Initial routine clinical genetic testing using targeted multigene panel sequencing did not identify pathogenic or likely pathogenic variants in *APC* or other polyposis‐associated genes. WES was performed as an additional investigation but also failed to reveal causative variants, prompting further analysis using WGS.

### 3.2. Kindred 1: Identification and Transcript‐Level Validation of a Large *APC* Exon Deletion

#### 3.2.1. Clinical Description

The proband (IV‐5) was a 20‐year‐old female who initially presented with abdominal pain, mucus‐laden bloody stool, and weight loss. Colonoscopic examination revealed hundreds of adenomatous polyps distributed throughout the colon, with most polyps measuring 0.5–2.0 cm in diameter. Laboratory evaluation showed elevated serum amylase and lipase levels (approximately 2–3 times the upper limit of normal); however, no radiologic evidence of acute pancreatitis was observed on the abdominal computed tomography (CT) scan (Table 1).

At 32 years of age, she underwent laparoscopic TPC‐IPAA. Histopathological examination confirmed the presence of more than 100 colonic tubular adenomas without evidence of malignant transformation. Two years postoperatively, follow‐up abdominal CT demonstrated multiple intra‐abdominal desmoid tumors exceeding 10 cm in maximal diameter, for which treatment with meloxicam was initiated. No recurrence of adenomas was detected at the anastomosis site during follow‐up surveillance. Serum amylase and lipase levels remained mildly elevated without clinical signs of pancreatitis exacerbation.

A distantly related affected family member (IV‐10) was diagnosed with FAP at 20 years of age and similarly underwent laparoscopic TPC‐IPAA. Final pathological examination demonstrated multiple tubular adenomas with low‐grade dysplasia. Unlike the proband, IV‐10 exhibited no extracolonic manifestations and has remained free of disease recurrence under regular follow‐up.

The family history demonstrated multiple affected individuals with FAP and colorectal cancer across generations. Two second‐generation relatives (II‐1 and II‐3) were diagnosed with colorectal cancer and died at 60 and 54 years of age, respectively. In the third generation, individuals III‐5, III‐6, and III‐8 were diagnosed with FAP and underwent surgical resection at 27, 30, and 31 years of age, respectively, with pathological confirmation of colorectal cancer.

#### 3.2.2. Genomic Findings

**Figure 3:**
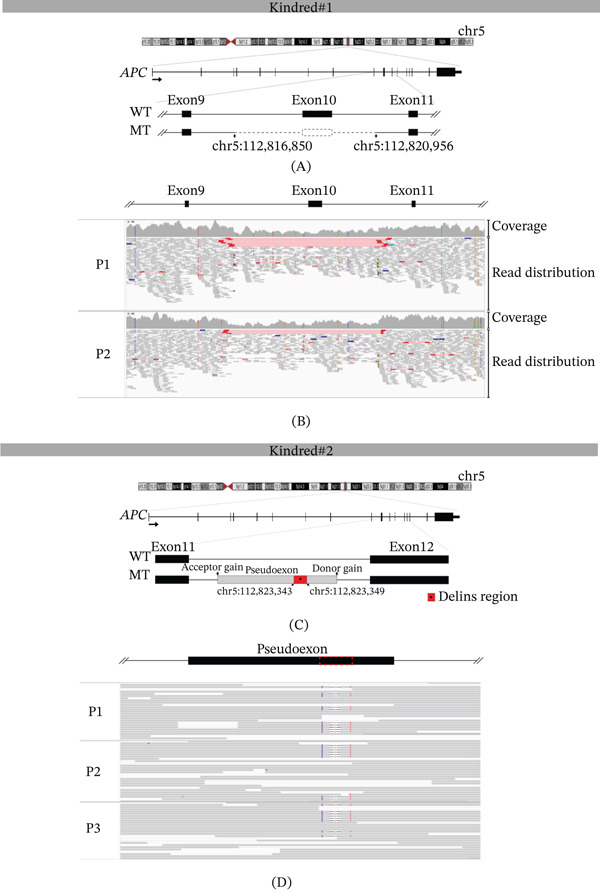
WGS identifies cryptic pathogenic *APC* variants missed by routine testing. (A) Schematic representation of the *APC* gene illustrating the location of a heterozygous large deletion encompassing exon 10 identified in Kindred 1. The deleted region is indicated as dashed lines. (B) Integrative Genomics Viewer (IGV) snapshots from Kindred 1 showing sequencing evidence supporting the exon 10–encompassing deletion. Reduced read depth across the deleted region is observed in affected individuals (P1 and P2), accompanied by multiple discordant paired‐end reads and split‐read alignments consistent with a structural deletion breakpoint. (C) Schematic overview of the deep intronic complex variant identified in Kindred 2, located within the intron between exons 11 and 12 of *APC*. The position of the variant is indicated as a red box, flanked by pseudoexon that is transcribed in a minor isoform (gray boxes). (D) IGV snapshots from Kindred 2 demonstrating the deep intronic *APC* variant consistently identified in affected family members (P1, P2, and P3), indicating a shared variant segregating with the disease phenotype. WT, wild type; MT, mutant.

In Kindred 1, WGS was performed on the proband (IV‐5) and a distantly related affected family member (IV‐10). Structural variant analysis using the Manta pipeline identified a heterozygous large deletion spanning approximately 4 kb within the *APC* gene, encompassing exon 10 (NC_000005.10(NM_000038.6):c.933+1257_1313‐940del) (Figure 3A). Visual inspection of sequencing data using the IGV demonstrated reduced read depth across the deleted region, accompanied by discordant paired‐end reads and split‐read alignments consistent with a structural deletion breakpoint (Figure 3B).

An additional known pathogenic variant in *SPINK1* (c.194+2T>C), previously associated with hereditary pancreatitis, was identified in the proband (IV‐5). This individual, along with an affected first‐degree relative (III‐5), who also carried the *SPINK1* variant, had a clinical history of recurrent pancreatitis. In contrast, this variant was not detected in IV‐10 (P2), who had no clinical history of pancreatitis and showed no laboratory evidence of elevated serum amylase and lipase levels. Although unrelated to the FAP phenotype, the co‐occurrence of a clinically relevant SPINK1 variant illustrates the added value of comprehensive genome‐wide testing.

#### 3.2.3. Transcript‐Level Validation

**Figure 4:**
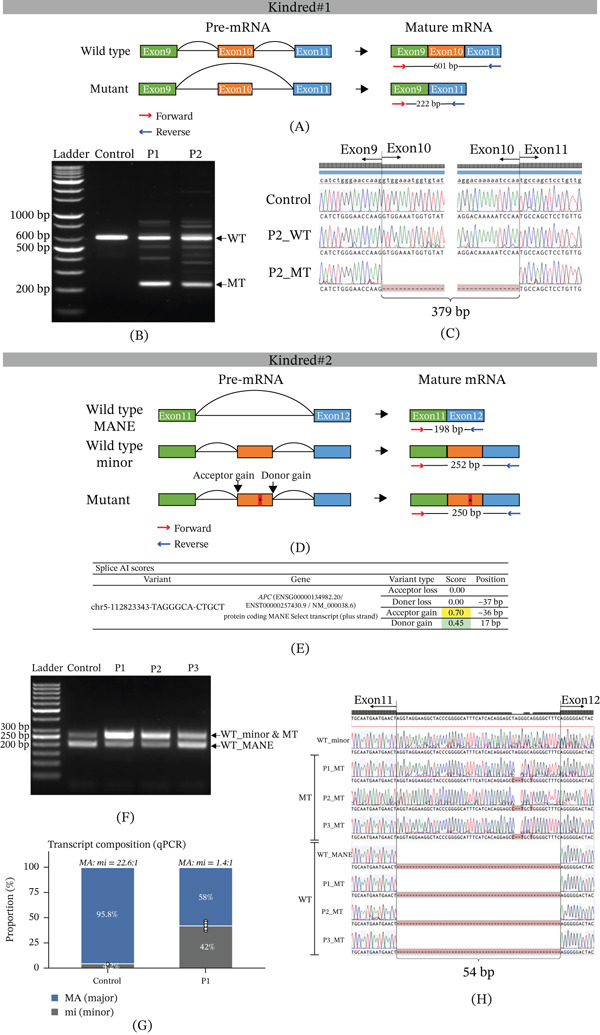
Transcript alterations and RNA‐level validation of cryptic *APC* variants. (A–C) Transcript‐level consequence of the exon 10–encompassing deletion identified in Kindred 1. (A) Schematic illustration showing exon 10 skipping resulting from the genomic deletion. (B) Agarose gel electrophoresis of RT‐PCR products spanning exons 9–11 of *APC* demonstrating an aberrant shorter transcript in affected individuals compared with controls. (C) Sanger sequencing chromatograms confirming direct splicing of exons 9–11, consistent with the complete loss of exon 10. (D–H) Aberrant splicing caused by a deep intronic *APC* variant identified in Kindred 2. (D) Schematic representation of pseudoexon inclusion resulting from activation of cryptic splice site. The minor transcript isoform (ENST00000504915.2) observed in controls is shown for comparison. (E) In silico splice prediction (SpliceAI) supporting creation of novel splice acceptor and donor sites. Variant types with high scores are marked with color. (F) RT‐PCR analysis spanning exons 11–12 of *APC* showing a distinct aberrant transcript in affected individuals (P1, P2, and P3). WT_MANE, canonical MANE Select transcript band; WT minor & MT, minor transcript and mutant band. (G) RT‐qPCR quantification of major (MA) and minor (mi) transcript proportions in control and P1 samples, demonstrating a marked increase in the minor transcript fraction in the affected individual. (4.2% vs. 42.0%) Open circles indicate the mi proportion from individual replicates (control, *n* = 4 replicates across two independent runs; P1, *n* = 6 replicates across three independent runs). (H) Sanger sequencing confirming inclusion of an aberrant pseudoexon harboring the 2‐base‐pair deletion in the mature transcript.

To evaluate the transcript‐level consequence of the deletion, RT‐PCR was performed using primers spanning exons 9–11 of *APC*. Gel electrophoresis revealed an aberrant shorter PCR product in affected individuals compared with the expected wild‐type amplicon, whereas no aberrant band was observed in healthy controls (Figure 4A,B, S1A). Sanger sequencing of the aberrant PCR product confirmed loss of exon 10 (Figure 4C, S1B).

#### 3.2.4. Predicted Protein Consequence

**Figure 5:**
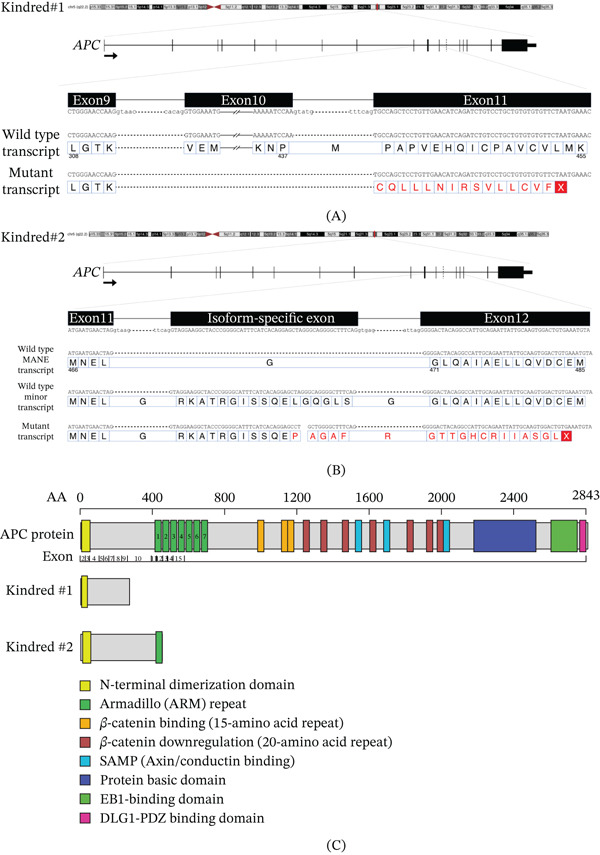
Predicted protein consequences of cryptic *APC* variants. (A) Predicted effect of the exon 10–encompassing deletion identified in Kindred 1 on the APC protein. Skipping of exon 10 results in a frameshift and introduction of a premature termination codon, leading to truncation of the APC protein with loss of multiple downstream functional domains essential for *β*‐catenin regulation. (B) Predicted protein consequence of aberrant pseudoexon inclusion caused by the deep intronic complex variant in Kindred 2. Activation of cryptic splice sites results in a frameshift and premature termination codon, producing a truncated APC protein consistent with a loss‐of‐function pathogenic mechanism. (C) Schematic representation of the full‐length APC protein and its functional domains, with comparison to the predicted truncated proteins in Kindred 1 and Kindred 2. Both variants result in premature truncation upstream of key functional domains, including *β*‐catenin–binding and regulatory regions, highlighting a shared loss‐of‐function mechanism despite distinct underlying mutational events.

The exon 10 deletion resulted in a frameshift predicted to introduce a premature termination codon downstream of the deletion (NC_000005.10(NP_000029.2):p.(Val312CysfsTer16)). The truncated APC protein is expected to lack multiple functional domains essential for *β*‐catenin regulation, consistent with a loss‐of‐function disease mechanism (Figure 5A,C).

### 3.3. Kindred 2: Deep Intronic *APC* Variant Causing Pseudoexon Activation

#### 3.3.1. Clinical Description

The proband (III‐2) was a 31‐year‐old male who presented with left lower quadrant abdominal pain and diarrhea. Colonoscopic examination revealed numerous adenomatous polyps throughout the colon and rectum. His family history was notable for colorectal cancer affecting multiple relatives across generations: His maternal grandmother was diagnosed with colorectal cancer and died at approximately 60 years of age, whereas his mother and maternal aunt were diagnosed with colorectal cancer in their early forties and subsequently underwent surgical resection. In addition, the proband′s maternal aunt′s son and daughter were diagnosed with FAP in their late twenties and also underwent surgical treatment.

At 32 years of age, the proband underwent laparoscopic TPC‐IPAA. Histopathological examination confirmed more than 100 colonic tubular adenomas without malignant transformation. Postoperative surveillance with colonoscopy and abdominal CT scan showed no evidence of adenoma recurrence, and the patient remains under regular outpatient follow‐up (Table 1).

#### 3.3.2. Genomic Findings

In Kindred 2, WGS identified a shared deep intronic complex variant within the intron between exons 11 and 12 of *APC* in affected family members. Initial variant calling using GATK HaplotypeCaller detected multiple adjacent intronic alterations in this region, including a duplication, a deletion, and a single‐nucleotide variant. As these changes occurred on the same haplotype, they were described as a single complex delins variant according to HGVS recommendations (NC_000005.10(NM_000038.6):c.1408+1352_1408+1358delinsCTGCT) (Figure 3C,D).

#### 3.3.3. In Silico Splice Prediction

In silico analysis, using SpliceAI predicted the creation of novel splice acceptor and donor sites at the variant locus, suggesting the potential activation of a cryptic exon. Notably, this genomic region corresponded to an exon present only in a known minor *APC* transcript isoform (ENST00000504915.2), raising the possibility of aberrant pseudoexon inclusion in affected individuals (Figure 4E).

#### 3.3.4. Transcript‐Level Validation

To assess the predicted splicing abnormality, RT‐PCR was performed using primers spanning exons 11–12 of *APC*. In control samples, a 198‐bp amplicon corresponding to the canonical MANE Select *APC* transcript was predominant, with a faint 252‐bp product corresponding to the known minor transcript isoform (ENST00000504915.2) observed at low abundance. In contrast, samples from affected individuals demonstrated a distinct aberrant upper band of approximately 250 bp, accompanied by a relative reduction of the canonical MANE transcript band (Figure 4F). Sanger sequencing of the aberrant product confirmed inclusion of a pseudoexon harboring a 2‐bp deletion, consistent with activation of cryptic splice sites predicted by in silico analysis (Figure 4H).

To quantitatively assess the shift in *APC* transcript composition, RT‐qPCR was performed using isoform‐specific primer pairs targeting the MA and mi transcripts, with *GAPDH* as a reference gene. The relative proportion of each transcript was calculated using the 2^(−*Δ*Ct)^ method. In control samples (*n* = 4 replicates across two independent runs), MA and mi comprised 95.8*%* ± 0.3*%* and 4.2*%* ± 0.3*%* of total *APC* expression, respectively (MA:mi ratio = 22.6 ± 1.6:1). In affected individuals from Kindred 2 (*n* = 6 replicates across three independent runs), the mi fraction increased markedly to 42.0*%* ± 3.5*%*, with a corresponding decrease in MA to 58.0*%* ± 3.5*%* (MA:mi ratio = 1.40 ± 0.20:1), representing an approximately 10‐fold increase in minor transcript abundance relative to controls (Figure 4G).

#### 3.3.5. Predicted Protein Consequence

Inclusion of the aberrant pseudoexon resulted in a frameshift predicted to introduce a premature termination codon. The resulting truncated *APC* protein is expected to be functionally impaired, consistent with a loss‐of‐function pathogenic mechanism (Figure 5B,C).

### 3.4. Variant Classification According to ACMG Guidelines

Both identified *APC* variants were classified as pathogenic according to the American College of Medical Genetics and Genomics and the Association for Molecular Pathology guidelines (Table 2).

**Table 2:** ACMG/AMP classification of pathogenic *APC* variants identified in this study.

Kindred	Gene	Variant (HGVS, MANE Select)	Variant type	ACMG evidence code	Evidence description	Classification
Kindred 1	*APC*	NM_000038.6:c.933+1257_1313‐940del	Large genomic deletion (exon‐level)	PVS1	Exon 10–encompassing deletion resulting in exon skipping, frameshift, and premature termination codon in a gene where loss of function is a known disease mechanism	Pathogenic
		NP_000029.2:p.(Val312CysfsTer16)				
				PS3	Transcript‐level functional studies demonstrated complete skipping of exon 10 by RT‐PCR and Sanger sequencing	
				PM2	Absent from population databases (gnomAD)	
				PP1	Variant segregates with the disease phenotype in multiple affected family members	
Kindred 2	*APC*	NM_000038.6:c.1408+1352_1408+1358delinsCTGCT	Deep intronic complex delins variant	PVS1	Aberrant splicing resulting in pseudoexon inclusion, frameshift, and premature termination codon	Pathogenic
				PS3	Functional transcript analysis confirmed pseudoexon activation by RT‐PCR and Sanger sequencing	
				PM2	Absent from population databases (gnomAD)	
				PP1	Variant segregates with disease phenotype in affected family members	

Abbreviations: ACMG, American College of Medical Genetics and Genomics; AMP, Association for Molecular Pathology; gnomAD, Genome Aggregation Database; MANE, Matched Annotation from NCBI and EMBL‐EBI.

In Kindred 1, the exon 10–encompassing deletion represents a null variant in a gene for which loss of function is an established disease mechanism (PVS1). Pathogenicity was further supported by absence from population databases (PM2), segregation with disease within the family (PP1), and transcript‐level functional evidence demonstrating exon skipping and a frameshift leading to premature termination (PS3).

In Kindred 2, although the variant was located in a deep intronic region, in silico predictions supported the creation of novel splice sites, and transcript‐level functional studies confirmed aberrant pseudoexon inclusion resulting in a frameshift and premature termination codon, fulfilling the criteria for PVS1 and PS3. Additional supporting evidence included absence from population databases (PM2) and segregation with the disease phenotype (PP1).

## 4. Discussion

In this study, we identified two cryptic pathogenic *APC* variants in clinically diagnosed FAP families in whom routine genetic testing had failed to establish a molecular diagnosis. By applying pedigree‐based WGS followed by transcript‐level functional validation, we demonstrated that both variants resulted in loss‐of‐function effects through distinct molecular mechanisms: a large exon‐level deletion and a deep intronic splice‐altering variant leading to pseudoexon activation.

A key finding of this study is the limitation of routine targeted panel sequencing—and even WES—in detecting certain classes of pathogenic *APC* variants. Although targeted panels are effective for identifying single‐nucleotide variants and small insertions or deletions within coding regions, they are inherently limited in their ability to detect exon‐level deletions and structural variants with intronic breakpoints [22, 31]. Similarly, WES may fail to identify such variants due to uneven coverage, capture design constraints, and the absence of reliable exon‐spanning structural variant detection. In Kindred 1, the exon 10–encompassing deletion involved intronic breakpoint regions that were poorly represented in exome capture, explaining its absence in prior analyses and underscoring the diagnostic advantage of WGS.

Beyond structural variants, our findings also highlight the diagnostic challenge posed by deep intronic splice‐altering variants. In Kindred 2, the identified variant resided outside canonical splice junctions and would not have been prioritized by conventional variant filtering strategies. Importantly, this variant activated cryptic splice acceptor and donor sites, resulting in the inclusion of a pseudoexon and a frameshift leading to premature termination. Notably, the affected genomic region corresponded to an exon present only in a minor *APC* transcript isoform under normal conditions. Functional transcript analysis revealed that this normally low‐abundance isoform became aberrantly expressed in affected individuals, effectively converting a minor transcript into a pathogenic one. RT‐qPCR quantification further demonstrated an approximately 10‐fold increase in the minor transcript fraction in the affected individual (MA:mi ratio 1.40:1) compared with controls (22.6:1), providing objective quantitative evidence of aberrant pseudoexon activation.

This observation underscores the importance of transcript‐aware variant interpretation, particularly in genes such as *APC* with complex transcript architectures [32]. Although the MANE Select transcript provides a standardized framework for variant annotation, exclusive reliance on a single reference transcript may obscure disease‐relevant splicing mechanisms. Our results demonstrate that pathogenicity may arise not only from direct disruption of the canonical transcript but also from aberrant activation of alternative transcript structures, emphasizing the need for RNA‐level validation in unresolved cases [33].

Both variants identified in this study resulted in frameshift events and premature termination codons, consistent with loss‐of‐function as the established disease mechanism in *APC*‐associated FAP. The combination of segregation analysis, absence from population databases, and direct transcript‐level functional evidence allowed confident classification of both variants as pathogenic according to ACMG guidelines [34]. In particular, functional RNA evidence (PS3) played a decisive role in resolving the pathogenicity of the deep intronic variant, which would otherwise remain challenging to interpret based solely on genomic data [35, 36].

From a clinical perspective, our findings have important implications for the diagnostic evaluation of patients with clinically suspected FAP who test negative on routine genetic screening. In such cases, WGS should be considered to identify cryptic structural or intronic variants, and transcript‐level analysis should be pursued when splicing abnormalities are suspected. This integrated approach not only increases diagnostic yield but also provides mechanistic insights that may inform genetic counseling and family screening.

Several limitations should be acknowledged. Transcript analyses were performed using RNA derived from peripheral blood, which may not fully reflect tissue‐specific splicing patterns in the colonic epithelium. Nevertheless, the clear demonstration of aberrant splicing and frameshift events in blood‐derived transcripts supports the pathogenic relevance of the identified variants. Future studies incorporating tissue‐specific transcriptomics or long‐read sequencing approaches may further refine our understanding of complex *APC* splicing mechanisms.

In conclusion, this study illustrates that cryptic pathogenic *APC* variants beyond the detection limits of routine genetic testing can be successfully identified through WGS and resolved through transcript‐level functional validation. Our findings reinforce the necessity of integrating genome‐wide sequencing and RNA‐based analyses into the diagnostic workflow for genetically unexplained FAP and highlight the broader relevance of transcript‐aware interpretation in hereditary cancer genetics.

## Funding

This study was supported by Lee Kun‐Hee Seoul National University Hospital Child Cancer & Rare Disease Project (26B‐001‐0500); MD‐PhD/Medical Scientist Training Program.

## Conflicts of Interest

The authors declare no conflicts of interest.

## Supporting information


**Supporting Information** Additional supporting information can be found online in the Supporting Information section. Figure S1 presents transcript‐level validation of the APC exon 10 deletion in three additional affected individuals from Kindred 1, including RT‐PCR gel electrophoresis and Sanger sequencing chromatograms confirming skipping of exon 10 and direct splicing from exons 9–11. Table S1 summarizes the quality metrics of the whole genome sequencing data generated in this study. Table S2 shows the primer sequences used for RT‐qPCR.

## Data Availability

The data that support the findings of this study are available on request from the corresponding author. The data are not publicly available due to privacy or ethical restrictions.

## References

[1] Vasen H. F. A. , Möslein G. , Alonso A. , Aretz S. , Bernstein I. , Bertario L. , Blanco I. , Bülow S. , Burn J. , Capella G. , Colas C. , Engel C. , Frayling I. , Friedl W. , Hes F. J. , Hodgson S. , Järvinen H. , Mecklin J. P. , Møller P. , Myrhøi T. , Nagengast F. M. , Parc Y. , Phillips R. , Clark S. K. , de Leon M. P. , Renkonen-Sinisalo L. , Sampson J. R. , Stormorken A. , Tejpar S. , Thomas H. J. W. , and Wijnen J. , Guidelines for the Clinical Management of Familial Adenomatous Polyposis (FAP), Gut. (2008) 57, no. 5, 704–713, 10.1136/gut.2007.136127. 18194984

[2] Jasperson K. W. , Tuohy T. M. , Neklason D. W. , and Burt R. W. , Hereditary and Familial Colon Cancer, Gastroenterology. (2010) 138, no. 6, 2044–2058, 10.1053/j.gastro.2010.01.054, 20420945. 20420945 PMC3057468

[3] Groden J. , Thliveris A. , Samowitz W. , Carlson M. , Gelbert L. , Albertsen H. , Joslyn G. , Stevens J. , Spirio L. , Robertson M. , Sargeant L. , Krapcho K. , Wolff E. , Burt R. , Hughes J. P. , Warrington J. , McPherson J. , Wasmuth J. , le Paslier D. , Abderrahim H. , Cohen D. , Leppert M. , and White R. , Identification and Characterization of the Familial Adenomatous Polyposis Coli Gene, Cell. (1991) 66, no. 3, 589–600, 10.1016/0092-8674(81)90021-0. 1651174

[4] Half E. , Bercovich D. , and Rozen P. , Familial Adenomatous Polyposis, Orphanet Journal of Rare Diseases. (2009) 4, no. 1, 10.1186/1750-1172-4-22, 19822006. PMC2772987 19822006

[5] Nishisho I. , Nakamura Y. , Miyoshi Y. , Miki Y. , Ando H. , Horii A. , Koyama K. , Utsunomiya J. , Baba S. , Hedge P. , Markham A. , Krush A. J. , Petersen G. , Hamilton S. R. , Nilbert M. C. , Levy D. B. , Bryan T. M. , Preisinger A. C. , Smith K. J. , Su L. K. , Kinzler K. W. , and Vogelstein B. , Mutations of Chromosome 5q21 Genes in FAP and Colorectal Cancer Patients, Science. (1991) 253, no. 5020, 665–669, 10.1126/science.1651563, 1651563. 1651563

[6] Morales J. , Pujar S. , Loveland J. E. , Astashyn A. , Bennett R. , Berry A. , Cox E. , Davidson C. , Ermolaeva O. , Farrell C. M. , Fatima R. , Gil L. , Goldfarb T. , Gonzalez J. M. , Haddad D. , Hardy M. , Hunt T. , Jackson J. , Joardar V. S. , Kay M. , Kodali V. K. , McGarvey K. M. , McMahon A. , Mudge J. M. , Murphy D. N. , Murphy M. R. , Rajput B. , Rangwala S. H. , Riddick L. D. , Thibaud-Nissen F. , Threadgold G. , Vatsan A. R. , Wallin C. , Webb D. , Flicek P. , Birney E. , Pruitt K. D. , Frankish A. , Cunningham F. , and Murphy T. D. , A Joint NCBI and EMBL-EBI Transcript Set for Clinical Genomics and Research, Nature. (2022) 604, no. 7905, 310–315, 10.1038/s41586-022-04558-8, 35388217. 35388217 PMC9007741

[7] Kinzler K. W. and Vogelstein B. , Lessons From Hereditary Colorectal Cancer, Cell. (1996) 87, no. 2, 159–170, 10.1016/s0092-8674(00)81333-1. 8861899

[8] Clevers H. and Nusse R. , Wnt/*β*-Catenin Signaling and Disease, Cell. (2012) 149, no. 6, 1192–1205, 10.1016/j.cell.2012.05.012. 22682243

[9] Aoki K. and Taketo M. M. , Adenomatous Polyposis Coli (APC): A Multi-Functional Tumor Suppressor Gene, Journal of Cell Science. (2007) 120, no. 19, 3327–3335, 10.1242/jcs.03485, 17881494. 17881494

[10] Näthke I. S. , The Adenomatous Polyposis Coli Protein: The Achilles Heel of the Gut Epithelium, Annual Review of Cell and Developmental Biology. (2004) 20, no. 1, 337–366, 10.1146/annurev.cellbio.20.012103.094541, 15473844. 15473844

[11] Marchis D. E. , Laura M. , Tonelli F. , Quaresmini D. , Della-Morte D. , Silvestris F. , Guadagni F. , and Palmirotta R. , Desmoid Tumors in Familial Adenomatous Polyposis, Anticancer Research. (2017) 37, no. 7, 3357–3366, 10.21873/anticanres.11702. 28668823

[12] Kumamoto K. , Ishida H. , and Tomita N. , Recent Advances and Current Management for Desmoid Tumor Associated With Familial Adenomatous Polyposis, Journal of the Anus, Rectum and Colon. (2023) 7, no. 2, 38–51, 10.23922/jarc.2022-074, 37113586. 37113586 PMC10129359

[13] Desmoid Tumor Working Group , The Management of Desmoid Tumours: A Joint Global Consensus-Based Guideline Approach for Adult and Paediatric Patients, European Journal of Cancer. (2020) 127, 96–107, 10.1016/j.ejca.2019.11.013, 32004793. 32004793

[14] Choi J. S. , Kim J.-Y. , Ahn M.-J. , Song S. , Kim D. , Choi S. H. , Park Y.-S. , Kim T.-J. , Jo S. , Kim T.-H. , and Shim S. C. , Celecoxib Is the Only Nonsteroidal Anti-Inflammatory Drug to Inhibit Bone Progression in Spondyloarthritis, BMB Reports. (2025) 58, no. 3, 140–145, 10.5483/BMBRep.2024-0062, 39757202. 39757202 PMC11955732

[15] Zhang Z. , Liang S. , Wang D. , Liang S. , Li Y. , Wang B. , Jiang T. , Zhao G. , Zhang X. , and Banerjee S. , A Novel Pathogenic Single Nucleotide Germline Deletion in *APC* Gene in a Four Generation Chinese Family With Familial Adenomatous Polyposis, Scientific Reports. (2017) 7, no. 1, 12357, 10.1038/s41598-017-10395-x, 28955048. 28955048 PMC5617841

[16] Wang D. , Liang S. , Zhang Z. , Zhao G. , Hu Y. , Liang S. , Zhang X. , and Banerjee S. , A Novel Pathogenic Splice Acceptor Site Germline Mutation in Intron 14 of the *APC* Gene in a Chinese Family With Familial Adenomatous Polyposis, Oncotarget. (2017) 8, no. 13, 21327–21335, 10.18632/oncotarget.15570, 28423518. 28423518 PMC5400587

[17] Zhang Z. , Liang S. , Huang H. , Wang D. , Zhang X. , Wu J. , Chen H. , Wang Y. , Rong T. , Zhou Y. , and Banerjee S. , A Novel Pathogenic Large Germline Deletion in Adenomatous Polyposis Coli Gene in a Chinese Family With Familial Adenomatous Polyposis, Oncotarget. (2016) 7, no. 31, 50392–50400, 10.18632/oncotarget.10408, 27391059. 27391059 PMC5226590

[18] Kanth P. , Grimmett J. , Champine M. , Burt R. , and Jewel Samadder N. , Hereditary Colorectal Polyposis and Cancer Syndromes: A Primer on Diagnosis and Management, American Journal of Gastroenterology. (2017) 112, no. 10, 1509–1525, 10.1038/ajg.2017.212, 28786406. 28786406

[19] Basso G. , Bianchi P. , Malesci A. , and Laghi L. , Hereditary or Sporadic Polyposis Syndromes, Best Practice & Research. Clinical Gastroenterology. (2017) 31, no. 4, 409–417, 10.1016/j.bpg.2017.05.011. 28842050

[20] Lionel A. C. , Costain G. , Monfared N. , Walker S. , Reuter M. S. , Hosseini S. M. , Thiruvahindrapuram B. , Merico D. , Jobling R. , Nalpathamkalam T. , Pellecchia G. , Sung W. W. L. , Wang Z. , Bikangaga P. , Boelman C. , Carter M. T. , Cordeiro D. , Cytrynbaum C. , Dell S. D. , Dhir P. , Dowling J. J. , Heon E. , Hewson S. , Hiraki L. , Inbar-Feigenberg M. , Klatt R. , Kronick J. , Laxer R. M. , Licht C. , MacDonald H. , Mercimek-Andrews S. , Mendoza-Londono R. , Piscione T. , Schneider R. , Schulze A. , Silverman E. , Siriwardena K. , Snead O. C. , Sondheimer N. , Sutherland J. , Vincent A. , Wasserman J. D. , Weksberg R. , Shuman C. , Carew C. , Szego M. J. , Hayeems R. Z. , Basran R. , Stavropoulos D. J. , Ray P. N. , Bowdin S. , Meyn M. S. , Cohn R. D. , Scherer S. W. , and Marshall C. R. , Improved Diagnostic Yield Compared With Targeted Gene Sequencing Panels Suggests a Role for Whole-Genome Sequencing as a First-Tier Genetic Test, Genetics in Medicine: Official Journal of the American College of Medical Genetics. (2018) 20, no. 4, 435–443, 10.1038/gim.2017.119, 28771251. 28771251 PMC5895460

[21] Shirts B. H. , Salipante S. J. , Casadei S. , Ryan S. , Martin J. , Jacobson A. , Vlaskin T. , Koehler K. , Livingston R. J. , King M.-C. , Walsh T. , and Pritchard C. C. , Deep Sequencing With Intronic Capture Enables Identification of an *APC* Exon 10 Inversion in a Patient With Polyposis, Genetics in Medicine: Official Journal of the American College of Medical Genetics. (2014) 16, no. 10, 783–786, 10.1038/gim.2014.30, 24675673. 24675673 PMC4752361

[22] Bozsik A. , Butz H. , Grolmusz V. K. , Polgár C. , Patócs A. , and Papp J. , Genome Sequencing-Based Discovery of a Novel Deep Intronic *APC* Pathogenic Variant Causing Exonization, European Journal of Human Genetics. (2023) 31, no. 7, 841–845, 10.1038/s41431-023-01322-y, 36828923. 36828923 PMC10326037

[23] Cummings B. B. , Marshall J. L. , Tukiainen T. , Lek M. , Donkervoort S. , Foley A. R. , Bolduc V. , Waddell L. B. , Sandaradura S. A. , O’Grady G. L. , Estrella E. , Reddy H. M. , Zhao F. , Weisburd B. , Karczewski K. J. , O’Donnell-Luria A. H. , Birnbaum D. , Sarkozy A. , Hu Y. , Gonorazky H. , Claeys K. , Joshi H. , Bournazos A. , Oates E. C. , Ghaoui R. , Davis M. R. , Laing N. G. , Topf A. , Genotype-Tissue Expression Consortium , Kang P. B. , Beggs A. H. , North K. N. , Straub V. , Dowling J. J. , Muntoni F. , Clarke N. F. , Cooper S. T. , Bönnemann C. G. , and MacArthur D. G. , Improving Genetic Diagnosis in Mendelian Disease With Transcriptome Sequencing, Science Translational Medicine. (2017) 9, no. 386, eaal5209, 10.1126/scitranslmed.aal5209, 28424332. 28424332 PMC5548421

[24] Li D. , Tian L. , and Hakonarson H. , Increasing Diagnostic Yield by RNA-Sequencing in Rare Disease—Bypass Hurdles of Interpreting Intronic or Splice-Altering Variants, Annals of Translational Medicine. (2018) 6, no. 7, 10.21037/atm.2018.01.14, 29955586. PMC6015936 29955586

[25] Bong D. , Sohn J. , and Lee S.-J. V. , Brief Guide to RT-qPCR, Molecules and Cells. (2024) 47, no. 12, 100141, 10.1016/j.mocell.2024.100141, 39476972. 39476972 PMC11612376

[26] Vaz-Drago R. , Custódio N. , and Carmo-Fonseca M. , Deep Intronic Mutations and Human Disease, Human Genetics. (2017) 136, no. 9, 1093–1111, 10.1007/s00439-017-1809-4. 28497172

[27] Anna A. and Monika G. , Splicing Mutations in Human Genetic Disorders: Examples, Detection, and Confirmation, Journal of Applied Genetics. (2018) 59, no. 3, 253–268, 10.1007/s13353-018-0444-7, 29680930. 29680930 PMC6060985

[28] Han P. , Wei G. , Cai K. , Xiang X. , Deng W. P. , Li Y. B. , Kuang S. , Dong Z. , Zheng T. , Luo Y. , Liu J. , Guan Y. , Li C. , Dey S. K. , Liao Z. , and Banerjee S. , Identification and Functional Characterization of Mutations in LPL Gene Causing Severe Hypertriglyceridaemia and Acute Pancreatitis, Journal of Cellular and Molecular Medicine. (2020) 24, no. 2, 1286–1299, 10.1111/jcmm.14768. 31901151 PMC6991700

[29] Zhang R. , Chen S. , Han P. , Chen F. , Kuang S. , Meng Z. , Liu J. , Sun R. , Wang Z. , He X. , Li Y. , Guan Y. , Yue Z. , Li C. , Kumar Dey S. , Zhu Y. , and Banerjee S. , Whole Exome Sequencing Identified a Homozygous Novel Variant in CEP290 Gene Causes Meckel Syndrome, Journal of Cellular and Molecular Medicine. (2020) 24, no. 2, 1906–1916, 10.1111/jcmm.14887, 31840411. 31840411 PMC6991682

[30] Dai Y. , Liang S. , Dong X. , Zhao Y. , Ren H. , Guan Y. , Yin H. , Li C. , Chen L. , Cui L. , and Banerjee S. , Whole Exome Sequencing Identified a Novel DAG1 Mutation in a Patient With Rare, Mild and Late Age of Onset Muscular Dystrophy-Dystroglycanopathy, Journal of Cellular and Molecular Medicine. (2019) 23, no. 2, 811–818, 10.1111/jcmm.13979, 30450679. 30450679 PMC6349151

[31] Spier I. , Horpaopan S. , Vogt S. , Uhlhaas S. , Morak M. , Stienen D. , Draaken M. , Ludwig M. , Holinski-Feder E. , Nöthen M. M. , Hoffmann P. , and Aretz S. , Deep intronicAPCmutations Explain a Substantial Proportion of Patients With Familial or Early-Onset Adenomatous Polyposis, Human Mutation. (2012) 33, no. 7, 1045–1050, 10.1002/humu.22082. 22431159

[32] Cummings B. B. , Karczewski K. J. , Kosmicki J. A. , Seaby E. G. , Watts N. A. , Singer-Berk M. , Mudge J. M. , Karjalainen J. , Satterstrom F. K. , O’Donnell-Luria A. H. , Poterba T. , Seed C. , Solomonson M. , Alföldi J. , Genome Aggregation Database Production Team , Alföldi J. , Armean I. M. , Banks E. , Bergelson L. , Cibulskis K. , Collins R. L. , Connolly K. M. , Covarrubias M. , Cummings B. B. , Daly M. J. , Donnelly S. , Farjoun Y. , Ferriera S. , Francioli L. , Gabriel S. , Gauthier L. D. , Gentry J. , Gupta N. , Jeandet T. , Kaplan D. , Karczewski K. J. , Laricchia K. M. , Llanwarne C. , Minikel E. V. , Munshi R. , Neale B. M. , Novod S. , O’Donnell-Luria A. H. , Petrillo N. , Poterba T. , Roazen D. , Ruano-Rubio V. , Saltzman A. , Samocha K. E. , Schleicher M. , Seed C. , Solomonson M. , Soto J. , Tiao G. , Tibbetts K. , Tolonen C. , Vittal C. , Wade G. , Wang A. , Wang Q. , Ware J. S. , Watts N. A. , Weisburd B. , Whiffin N. , Genome Aggregation Database Consortium , Salinas C. A. A. , Ahmad T. , Albert C. M. , Ardissino D. , Atzmon G. , Barnard J. , Beaugerie L. , Benjamin E. J. , Boehnke M. , Bonnycastle L. L. , Bottinger E. P. , Bowden D. W. , Bown M. J. , Chambers J. C. , Chan J. C. , Chasman D. , Cho J. , Chung M. K. , Cohen B. , Correa A. , Dabelea D. , Daly M. J. , Darbar D. , Duggirala R. , Dupuis J. , Ellinor P. T. , Elosua R. , Erdmann J. , Esko T. , Färkkilä M. , Florez J. , Franke A. , Getz G. , Glaser B. , Glatt S. J. , Goldstein D. , Gonzalez C. , Groop L. , Haiman C. , Hanis C. , Harms M. , Hiltunen M. , Holi M. M. , Hultman C. M. , Kallela M. , Kaprio J. , Kathiresan S. , Kim B.-J. , Kim Y. J. , Kirov G. , Kooner J. , Koskinen S. , Krumholz H. M. , Kugathasan S. , Kwak S. H. , Laakso M. , Lehtimäki T. , Loos R. J. F. , Lubitz S. A. , Ma R. C. W. , MacArthur D. G. , Marrugat J. , Mattila K. M. , McCarroll S. , McCarthy M. I. , McGovern D. , McPherson R. , Meigs J. B. , Melander O. , Metspalu A. , Neale B. M. , Nilsson P. M. , O’Donovan M. C. , Ongur D. , Orozco L. , Owen M. J. , Palmer C. N. A. , Palotie A. , Park K. S. , Pato C. , Pulver A. E. , Rahman N. , Remes A. M. , Rioux J. D. , Ripatti S. , Roden D. M. , Saleheen D. , Salomaa V. , Samani N. J. , Scharf J. , Schunkert H. , Shoemaker M. B. , Sklar P. , Soininen H. , Sokol H. , Spector T. , Sullivan P. F. , Suvisaari J. , Tai E. S. , Teo Y. Y. , Tiinamaija T. , Tsuang M. , Turner D. , Tusie-Luna T. , Vartiainen E. , Vawter M. P. , Ware J. S. , Watkins H. , Weersma R. K. , Wessman M. , Wilson J. G. , Xavier R. J. , Daly M. J. , and MacArthur D. G. , Transcript Expression-Aware Annotation Improves Rare Variant Interpretation, Nature. (2020) 581, no. 7809, 452–458, 10.1038/s41586-020-2329-2, 32461655. 32461655 PMC7334198

[33] Walker L. C. , de la Hoya M. , Wiggins G. A. R. , Lindy A. , Vincent L. M. , Parsons M. T. , Canson D. M. , Bis-Brewer D. , Cass A. , Tchourbanov A. , Zimmermann H. , Byrne A. B. , Pesaran T. , Karam R. , Harrison S. M. , Spurdle A. B. , Biesecker L. G. , Harrison S. M. , Tayoun A. A. , Berg J. S. , Brenner S. E. , Cutting G. R. , Ellard S. , Greenblatt M. S. , Kang P. , Karbassi I. , Karchin R. , Mester J. , O’Donnell-Luria A. , Pesaran T. , Plon S. E. , Rehm H. L. , Strande N. T. , Tavtigian S. V. , and Topper S. , Using the ACMG/AMP Framework to Capture Evidence Related to Predicted and Observed Impact on Splicing: Recommendations From the ClinGen SVI Splicing Subgroup, American Journal of Human Genetics. (2023) 110, no. 7, 1046–1067, 10.1016/j.ajhg.2023.06.002, 37352859. 37352859 PMC10357475

[34] Richards S. , Aziz N. , Bale S. , Bick D. , das S. , Gastier-Foster J. , Grody W. W. , Hegde M. , Lyon E. , Spector E. , Voelkerding K. , Rehm H. L. , and ACMG Laboratory Quality Assurance Committee , Standards and Guidelines for the Interpretation of Sequence Variants: A Joint Consensus Recommendation of the American College of Medical Genetics and Genomics and the Association for Molecular Pathology, Genetics in Medicine: Official Journal of the American College of Medical Genetics. (2015) 17, no. 5, 405–424, 10.1038/gim.2015.30, 25741868. 25741868 PMC4544753

[35] Brnich S. E. , Abou Tayoun A. N. , Couch F. J. , Cutting G. R. , Greenblatt M. S. , Heinen C. D. , Kanavy D. M. , Luo X. , McNulty S. M. , Starita L. M. , and Tavtigian S. V. , Recommendations for Application of the Functional Evidence PS3/BS3 Criterion Using the ACMG/AMP Sequence Variant Interpretation Framework, Genome Medicine. (2019) 12, no. 1, 10.1186/s13073-019-0690-2, 31892348. PMC6938631 31892348

[36] Hong S. E. , Kneissl J. , Cho A. , Kim M. J. , Park S. , Lee J. , Woo S. , Kim S. , Kim J.-S. , Kim S. Y. , Jung S. , Kim J. , Shin J.-Y. , Chae J.-H. , and Choi M. , Transcriptome-Based Variant Calling and Aberrant mRNA Discovery Enhance Diagnostic Efficiency for Neuromuscular Diseases, Journal of Medical Genetics. (2022) 59, no. 11, 1075–1081, 10.1136/jmedgenet-2021-108307, 35387801. 35387801 PMC9613860

