# Supplementary material for: Identification of Novel Pathogenic Variants in Familial Adenomatous Polyposis Through Whole Genome Sequencing
Source: Hum Mutat. 2026 Aug 19;2026:1896928. doi: 10.1155/humu/1896928 (PMC13487699; doi:10.1155/humu/1896928)
Supplement: Supplementary file 1 [file HUMU-2026-1896928-s001.docx]

**<Supplementary Material>**

**Identification of Novel Pathogenic Variants in Familial Adenomatous Polyposis through Whole Genome Sequencing**

Sung-Ryung Choi^1,2^, Min Jung Kim^2,3^, Ja-Lok Ku^3,4,5,^, Seung-Yong Jeong^2,3^, Murim Choi^4^, Ji Won Park^2,3^

**Figure S1.Transcript-level validation of the *APC* exon 10 deletion in additional affected family members from Kindred 1**

(A) Agarose gel electrophoresis of RT-PCR products spanning exons 9 to 11 of *APC* obtained from three additional affected individuals in Kindred 1 (III-8, III-5, and IV-11). In all three individuals, both the wild-type band and a shorter aberrant band corresponding to exon 10 skipping are observed, demonstrating consistent aberrant splicing across affected family members.

(B) Sanger sequencing chromatograms of the aberrant RT-PCR products from individuals III-8, III-5, and IV-11. Direct splicing of exon 9 to exon 11 is confirmed in all samples, indicating complete loss of exon 10 at the transcript level. Shaded regions highlight the exon–exon junction corresponding to the aberrantly spliced transcript.

**Table S1. Sequencing quality metrics for whole genome sequencing**

|  | **Kindred 1** | | **Kindred 2** | | |
| --- | --- | --- | --- | --- | --- |
| **Sample** | P1 (IV-5) | P2 (IV-10) | P1 (III-2) | P2 (III-3) | P3 (III-4) |
| **Library prerp** | TruSeq Nano DNA | | | | |
| **Read length** | 151 bp PE | | | | |
| **Total reads** | 623.5 M | 598.0 M | 738.3 M | 639.2 M | 634.3 M |
| **Mapped reads** | 622.9 M (99.9%) | 597.5 M (99.9%) | 738.3 M (100.0%) | 632.9 M (100.0%) | 626.9 M (99.9%) |
| **Duplication rate** | 0.70% | 0.75% | 0.42% | 0.41% | 0.53% |
| **Mean coverage** | 30x | 29x | 26x | 23.5x | 21.7x |
| **% bases ≥ Q30** | 98.6% | 98.5% | 98.5% | 94.94% | 95.36% |
| **GC content** | 40.5% | 40.6% | 40.5% | 40.83% | 40.91% |

**Table S2. Primer sequences used for RT-qPCR**

| **Target** | **Direction** | **Sequence (5’ → 3’)** |
| --- | --- | --- |
| ***GAPDH*** | Forward | ATGGGGAAGGTGAAGGTCG |
|  | Reverse | TAAAAGCAGCCCTGGTGACC |
| ***APC* major transcript** | Forward | ATCCAATGCCAGCTCCTGTT |
|  | Reverse | CCCCTAGTTCATTCATTGCATGT |
| ***APC* minor transcript** | Forward | ATCCAATGCCAGCTCCTGTT |
|  | Reverse | GGGTAGCCTTCCTACCTAGTTCA |

*The major and minor transcript-specific primer pairs share an identical forward primer; transcript specificity is conferred by distinct reverse primers targeting the exon 11–12 canonical junction and the pseudoexon–exon 12 junction, respectively.*
